# Spatial and Annual Temporal Distribution of Ozone Concentrations in the Madrid Basin Using Passive Samplers

**DOI:** 10.1100/tsw.2001.316

**Published:** 2001-11-30

**Authors:** M.J. Sanz, F. Sanz, G. Sanchez-Peaa

**Affiliations:** Fundación CEAM, Parque Tecnológico, c/ Charles Darwin, 14, 46980 Valencia, Madrid, Spain

**Keywords:** ozone, passive sampler, Madrid

## Abstract

Passive samplers are useful tools for helping to describe the ozone distribution in complex terrain situations. They are also a good complement to continuous monitoring stations. This paper discusses the results of a pilot study that used ozone passive samplers to describe the spatial and annual temporal distribution of ozone in several forested areas around the city of Madrid. The ozone concentrations around Madrid were found to be higher on the elevated sites located at a certain distance from the city's urban zone. A seasonal ozone cycle was observed, with maximum concentrations found in the basin in late spring or summer depending on the location. The information obtained allowed us to group the locations into four classes. Altitude and distance to the city during the summer and winter explained the observed ozone concentrations. However, during the transition periods, especially in early spring and to a lesser extent in autumn, there was not a good correlation between ozone levels and elevation or distance from precursor sources. These data strongly suggest that altitudinal gradients for ozone are not always the case in the Madrid Basin.

## INTRODUCTION

Tropospheric ozone ($O_3$) is a major pollutant resulting from emissions from various sources, among them urban traffic through photochemical transformation of nitrogen oxides, carbon monoxide, and volatile organic compounds. Over the last 25 years, the Mediterranean Basin has become heavily industrialized, and, as a consequence, reports concerning air pollution damage in forested areas have increased since the late 1970s[1,2,3,4,5]. Now, it is well known that photo-oxidants, and especially ozone, are the most important pollutants in Mediterranean countries where the particular meteorological conditions are especially favourable to their formation and persistence[6]. In several regions of Spain, the dynamics of ozone and other pollutants have been documented within several EC-supported projects[6,7,8], showing that the horizontal distribution of air pollutants (i.e., ozone) is nonhomogeneous and that it undergoes seasonal variations and marked diurnal cycles in which mesoscale processes play an important role. In the western Mediterranean Basin the observed ozone cycles depend strongly on the topographic location of the observing station and its relationship to the reservoir layers, the atmospheric circulations involved, and the chemical processes along each path. The role of complex topography and associated meteorological processes in ozone distribution in mountain areas has been studied in several areas[9,10,11,12,13,14]. Most ozone monitoring efforts in complex terrain or remote areas have relied on electronic analysers that take continuous measurements. Thus, it can be assumed that in complex mountain areas each ozone monitoring station shows a part of the whole and could even be considered to represent a specific area, provided that the relevant processes are understood for each site and that the site itself has been adequately selected. However, no single station can be considered representative of regional processes, much less of the whole situation. The knowledge of these meteorological processes has been proven to provide useful information to interpret the patterns of distribution of the damage caused by air pollutants in forest ecosystems[15]. However, continuous monitors are costly to operate and require power and adequate space. It is quite common that there are large distances between continuous monitoring stations, making it very difficult to infer the spatial patterns of ozone in complex mountainous areas. Interest in passive monitors has recently been rekindled by the need to monitor ozone in remote forested areas where there is no access to power, and such monitors show numerous advantages[16]. For these reasons, passive samplers are useful tools to describe the ozone distribution in complex terrain situations. They are also a good complement to continuous monitoring stations. Passive samplers have been tested for other purposes such as use in human health studies[17] and in remote natural areas like Mount Rainer National Park[18] and the Carpathian Mountains[19].This paper discusses the results of a pilot study that used ozone passive samplers to describe the spatial and annual temporal distribution of ozone concentrations in several forested areas around the city of Madrid.

## MATERIAL AND METHODS

### Research Sites

The city of Madrid has a dense Air Pollution Monitoring Network with 24 automatic stations[20]. At each station the air is sampled at 2.5 m above the surface and the concentrations of $SO_2$, CO, NOx, $O_3$, HC (hydrocarbons), and particles are continuously determined. Since the network was designed for air quality control purposes following management criteria, its spatial coverage is largely confined to the city itself. It thus produces very limited information on areas in the vicinity of the city and the basin, i.e., the total Madrid air basin. This lack of air pollution data in suburban and rural areas is especially important when ozone is considered, because ozone levels are higher downwind of cities where high emissions of precursors are emitted[7], whereas the ozone concentration is depleted inside the city by the excess of precursors[21]. In order to explore the spatial distribution of ozone concentrations and the annual temporal ozone concentration distribution in the basin at certain distances from the city where forest and natural ecosystems are already present, research sites were established at 12 locations around the city of Madrid in spring 2000 (Table 1). Bulk deposition was also collected on the same locations for other purposes (data not shown).

**TABLE 1**
**Sampling Locations (1–12) and Continuous Monitoring Stations used for Validation and Correction of the Experimental Data**

| Location | Nº | Co-ordinates | Altitude (msm) | Main Tree Species |
|---|---|---|---|---|
| Villaviciosa de Odon | 1 | 40º22´11.2´´ N, 3º56´9.5´´ W | 670 | *Populus alba* |
| Navalgamella | 2 | 40º26´27´´ N, 4º 7´47´´ W | 830 | *Juniperus oxycedrus* |
| San Martín de Valdeiglesias | 3 | 40º18´37.5´´ N, 4º21´23.7´´ W | 900 | *Pinus pinea, Quercus ilex* |
| Galapagar | 4 | 40º34´5.9´´ N, 3º58´32.4´´ W | 930 | *P. sylvestris, P. pinaster* |
| El Escorial | 5 | 40º36´20.5´´ N, 4º9´21.4´´ W | 1360 | *P. sylvestris* |
| El Ventorrillo | 6 | 40º45´23.1´´ N, 4º1´18.3´´ W | 1530 | *Q. pyrenaica, Q. Ilex* |
| Miraflores de la Sierra | 7 | 40º48´13.4´´ N, 3º47´30.8´´ W | 1280 | *Q. pyrenaica* |
| Braojos | 8 | 41º3´50.6´´ N, 3º38´49.7´´ W | 1450 | *P. halepensis* |
| Belvis de Jarama | 9 | 40º33´45.4´´ N, 3º32´51.4´´ W | 650 | *Q. ilex* |
| Valdeaveruelo | 10 | 40º38´4.1´´ N, 3º18´37.6´´ W | 820 | *Q. ilex* |
| Belmonte del Tajo | 11 | 40º6´57.5´´ N, 3º19´15.8´´ W | 790 | *Q. ilex, P. halepensis* |
| La Marañosa | 12 | 41º31´8.8´´ N, 3º35´52.5´´ W | 698 | *P. halepensis* |
| Morella | M1 | 40º38´16.2´´N, 0º5´28.2´´ W | 1156 | *Q. ilex* |
| Gandía | M2 | 38º58´22.2´´ N, 0º9´58.5´´ W | 12 | - |
| Benifaió | M3 | 39º17´12.6´´ N, 0º26´30.3´´ W | 32 | *Citrus sinensis* |

## Ozone Monitoring

Average ambient concentrations of ozone were measured with Ogawa and Co., Inc. passive ozone samplers. Each sampler consists of a small Teflon cartridge containing two nitrite-coated filters. Air diffuses through steel mesh grids which hold the filters in place. In the presence of ozone, the nitrite is oxidized to nitrate, and after the exposure the filters are analysed for total nitrate concentration. The 2-week averages of ozone concentrations (ppbv) were calculated as suggested by Koutrakis et al.[22] . The samplers were exposed to ambient air over a 15-day period. The measurements were carried out from 15 April 2000 to the end of March 2001. At two stations of the Valencian Community Air Quality Network (M1, M2) and at the CEAM OTC experimental field (Benifaió, M3), ozone was measured with passive samplers accompanied by continuous monitors during the same time period. Ozone passive samplers at these locations were calibrated against Dasibi 1008RS ozone monitors that were, in turn, calibrated once a month. The results of these calibrations were used for correcting ozone concentrations for the remaining passive samplers in the Madrid network. Three blanks were shipped with the samplers to test for transport problems, and the samplers were then kept in the office of the DGCN (Ministry of the Environment) in Madrid during the exposure period.

## Data Analysis

The 2-week average ozone concentrations were calculated for each passive sampler location (ppbv). Sampler precision corresponded to the percent difference between replicate samplers at each site.

Sampler accuracy was determined by comparing passive sampler values with the continuous analyser hourly averages for identical sampling periods. Plotting both 2-week averages (from the monitors and the passive samplers), a linear regression equation was obtained (SPSS+), which was later used for corrections. Ozone passive sampler values were plotted against elevation and distance to the city to detect if there was a relationship between them (SPPS+). Independent simple linear regressions for each period and location for elevation and distance were calculated, as was a multiple linear regression including both variables to explore better fits.

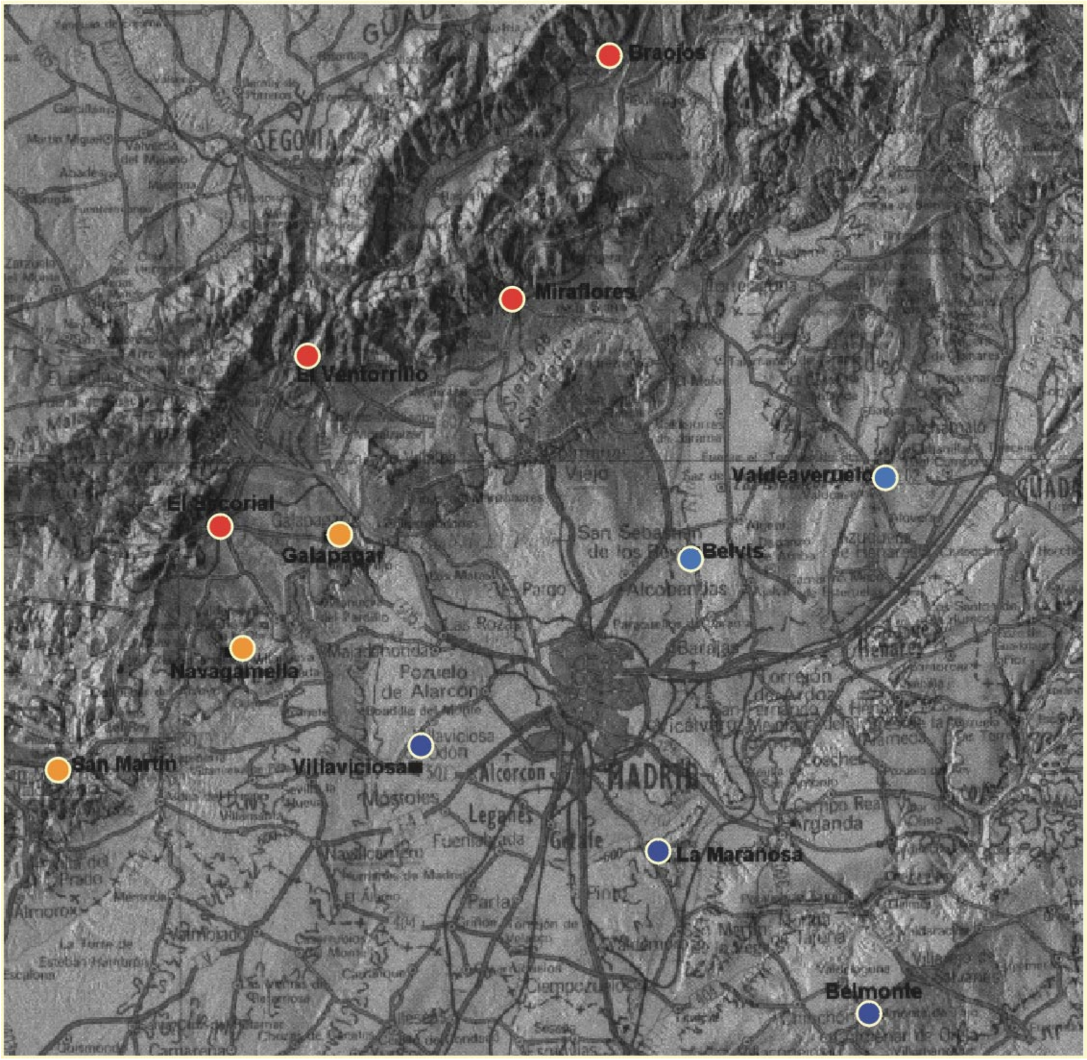

**FIGURE 1.** Map of the locations: Class I (red), Class II (orange), Class III (light blue), and Class IV (blue).

## RESULTS AND DISCUSSION

### Validation of the Ozone Passive Samplers

The mean ozone concentration determined from the blanks during the sampling period was 0.3159 ± 0.2520 ppbv. The precision of the pairs of ozone samplers was estimated as 1.6751 ppbv (maximum 14.7177 and minimum 0.0001), with an average variation coefficient of 6% during the study period (maximum 20% and minimum 0%). Linear regression analysis was used to compare ozone measurements by passive sampler and by continuous monitoring (Dasibi 1008RS). The regression equation gave a slope close to 1 (1.035 ± 0.020 ppbv) with a y-intercept of 0 (Fig. 2). The correlation coefficient for 30 measurements over the 2-week sampling periods was $r^2 = 0.995$ and was evenly distributed over the study period. Data were corrected using the equation to produce the final results.

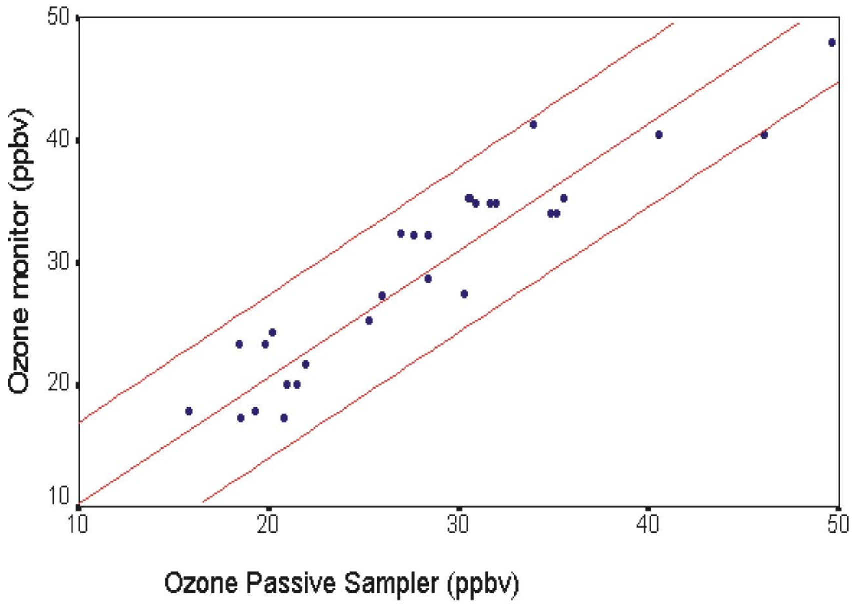

$r^2 = 0.9946$ Y = 1.035 X    SE = 0.020

**FIGURE 2.** Linear regression between the passive sampling and the continuous monitor concentrations for locations M1, M2, and M3. Lines indicate 95% confidence interval.

## Spatial Distribution of Ozone Within the Basin

The passive sampler locations were grouped into four classes as a function of the distance to the downtown urban area and the altitude. The 2-week average ozone concentrations generally increased with altitude and distance away from the urban area, and determined the class. Class I (Fig. 3) comprises locations 4 to 8. These were locations with altitudes higher than 1000 msm and at a certain distance from the urban area; they are on the south-facing slopes of Madrid's northwest mountains, where the highest photochemical oxidant production was expected. Class II (Fig. 4) comprises the locations that are in the "Rampa" of Madrid: smooth, prolonged slopes facing south at the southwest of Madrid and located closer to the city. Classes III (Fig. 5, locations 9 and 10) and IV (Fig. 6, locations 1, 11, and 12) are locations on the bottom of the basin, north and south-southwest of the city respectively, where precursor emissions are still very high and/or ozone formation has not yet taken place completely. Maximum values were found on Class I locations, with the highest value being 71 ppbv in Braojos (location 8). The lowest ozone value was 47 ppbv in Villaviciosa (location 1, Class IV). Class I location values oscillated between 14.5 and 71 ppbv. Class II locations varied between 11.7 and 63 ppbv, Class III between 8.5 and 63 ppbv, and Class IV between 8.5 and 51.8 ppbv. The maximum 2-week ozone values observed are considered high to moderately high when compared with other areas such as the east coast of Spain (Sanz, data not published).

## Temporal Evolution of Ozone Concentrations Throughout the Year

During the study period considered, two main ozone peaks were observed at all locations: one in late spring and one in midsummer with a temporal decay in July (Figs. 3 to 6). The same type of behaviour has been observed at other monitoring networks on the east coast of Spain[14] where only electronic monitors are used. Thus, passive ozone sampling appears to be a useful tool for studying the annual temporal variation in ozone concentrations over the year when 2-week

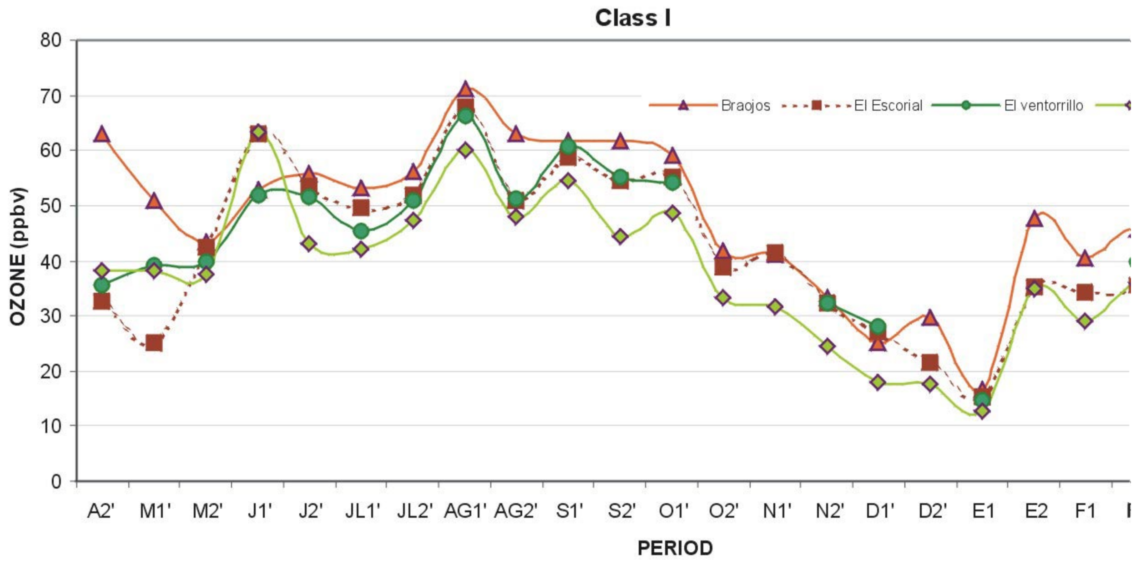

**FIGURE 3.** Ozone 2-week averages (ppbv) for all Class I locations from mid-April 2000 (April 2) to end of March 2001 (March 2).

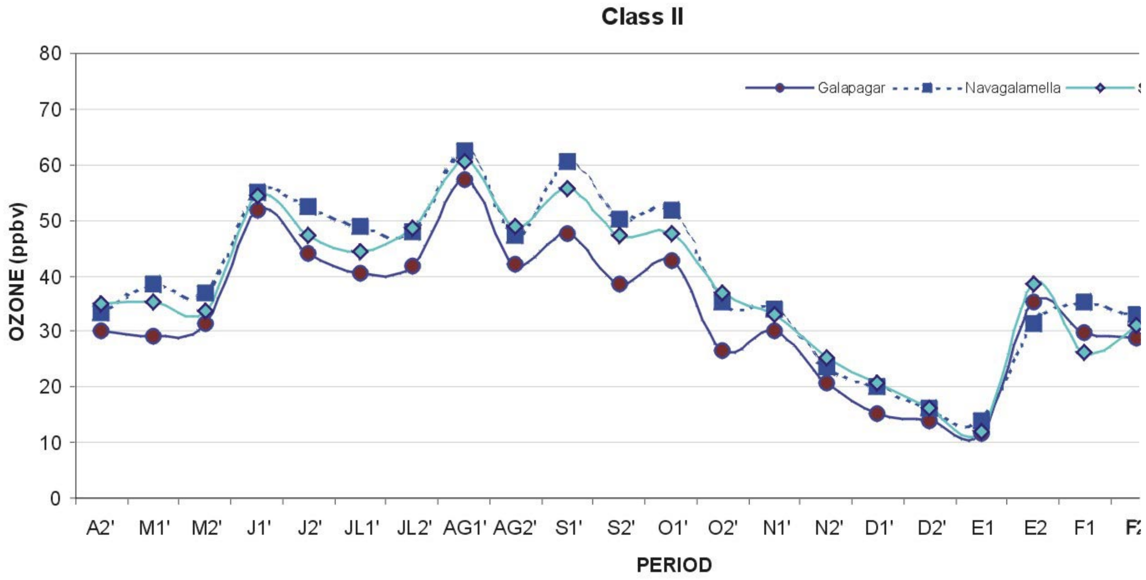

**FIGURE 4.** Ozone 2-week averages (ppbv) for all the Class II locations from mid-April 2000 (April 2) to end of March 2001 (March 2).

periods are considered. Class I to III locations show higher values in midsummer than in late spring, except for Belvis (Class III). This location is close enough to Madrid and presents an elevation low enough (650 m.s.m) to be considered a transition location between Classes III and IV or to be included in class IV. On the other hand, Class IV stations (locations 1, 11, and 12) show an absolute maximum in late spring instead of midsummer.

For all the passive sampler locations, minimum ozone values are found during winter, in January, as expected. Values ranged from 8.5 to 16.8 ppbv, with the Class I locations showing the highest values . The high ozone values observed at high elevations probably result from the fact that such locations are influenced most of the time by the reservoir layers generated by valley recirculations or long-range transport[7]. In fact, the presence of atmospheric layers with tem-

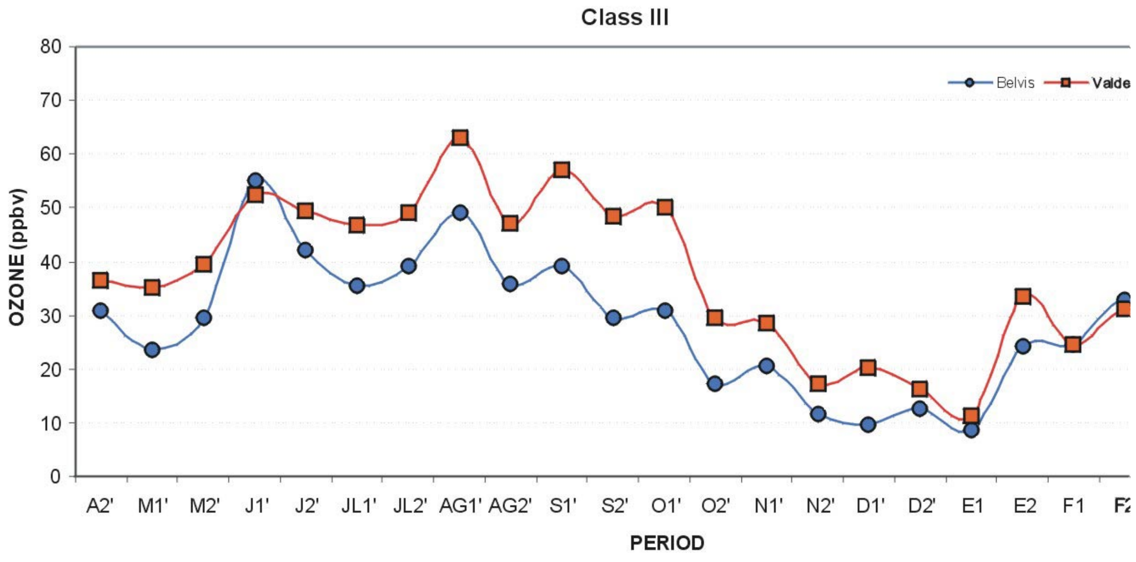

**FIGURE 5.** Ozone 2-week averages (ppbv) for all the Class III locations from mid-April 2000 (April 2) to end of March 2001 (March 2).

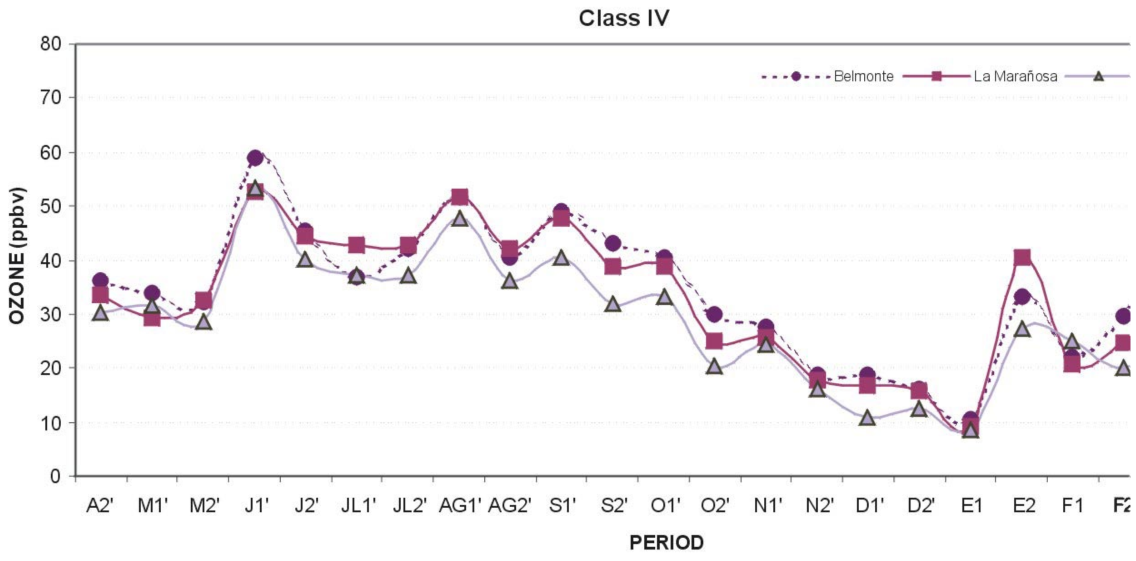

**FIGURE 6.** Ozone 2-week averages (ppbv) for all the Class IV locations from mid-April 2000 (April 2) to end of March 2001 (March 2).

peratures close to the adiabatic lapse rate was detected in other studies over the Madrid Basin in July 1989[7], July 1994, and 1995[23]. Thus, the decoupling between upper and lower stations may occur more often in winter when the altitude of the inversion layer is lower. During the warmer seasons, a combination of two types of processes may be responsible for the gradients observed: (1) photochemical production with increasing distance from the source of precursors[21] combined with local transport of the air mass within the basin as a result of the recirculations[14], and (2) the existence of reservoir layers aloft generated during previous days or long-range transport[6]. Topography drives the circulation patterns in the basin, with the strength of the up-slope winds dependent on solar radiation heating of the slopes and the properties of the surface during the day. At night, down-slope drainage flows are established. Depending on the valley or basin aspect, the cold air can accumulate on the bottom or keep flowing toward other areas[24]. Thus, the correlation between the 2-week ozone averages and elevation is not always good over the entire year (Table 2) because the processes mentioned

**TABLE 2**
**Slope, Origin Ordinate, and Regression Coefficient ( $r^2$) between Ozone 2-Week Average Distance and Elevation***

| | Regressions – Elevation | | | Regressions – Distance to the City | | | Multiple Regression | | | |
|---|---|---|---|---|---|---|---|---|---|---|
| Period | Slope a | Origin ordinate c | $r^2$ | Slope b | Origin ordinate c | $r^2$ | Slope a | Slope b | Origin ordinate c | $r^2$ |
| April 2 | 0.0146 | 21.784 | 0.2823 | 0.4054 | 20.266 | 0.5339 | -0.0010 | 0.4212 | 20.670 | 0.7313 |
| May 1 | 0.0118 | 22.418 | 0.2703 | 0.3508 | 20.336 | 0.5802 | -0.0027 | 0.3911 | 21.390 | 0.7656 |
| May 2 | 0.0127 | 23.048 | 0.6668 | 0.2404 | 26.154 | 0.5859 | 0.0086 | 0.1099 | 22.756 | 0.8490 |
| June 1 | 0.0011 | 57.145 | 0.0009 | 0.0389 | 53.892 | 0.0277 | 0.0045 | -0.0297 | 52.114 | 0.2782 |
| June 2 | 0.0102 | 37.325 | 0.4211 | 0.2376 | 38.094 | 0.5571 | 0.0032 | 0.1890 | 36.829 | 0.7585 |
| July 1 | 0.0108 | 32.948 | 0.3921 | 0.2442 | 34.030 | 0.4908 | 0.0040 | 0.1840 | 34.464 | 0.7168 |
| July 2 | 0.0141 | 32.215 | 0.1401 | 0.3115 | 33.965 | 0.7896 | 0.0059 | 0.2218 | 31.628 | 0.8993 |
| August 1 | 0.0195 | 39.709 | 0.6833 | 0.3977 | 43.431 | 0.6913 | 0.0110 | 0.2310 | 39.103 | 0.8863 |
| August 2 | 0.0189 | 27.357 | 0.6563 | 0.4236 | 29.416 | 0.8057 | 0.0073 | 0.3129 | 26.535 | 0.9212 |
| September 1 | 0.0176 | 35.292 | 0.5231 | 0.4090 | 36.639 | 0.6903 | 0.0056 | 0.3244 | 34.443 | 0.8445 |
| September 2 | 0.0237 | 21.797 | 0.6294 | 0.5357 | 24.195 | 0.7863 | 0.0088 | 0.4027 | 20.737 | 0.9078 |
| October 1 | 0.0224 | 23.858 | 0.6584 | 0.4761 | 27.310 | 0.7276 | 0.0108 | 0.3117 | 23.036 | 0.8916 |
| October 2 | 0.0216 | 10.053 | 0.6413 | 0.4363 | 13.653 | 0.8462 | 0.0062 | 0.3524 | 11.016 | 0.9317 |
| November 1 | 0.0199 | 11.871 | 0.7655 | 0.3287 | 18.051 | 0.6720 | 0.0136 | 0.1448 | 12.267 | 0.9050 |
| November 2 | 0.0200 | 2.8942 | 0.8392 | 0.3633 | 8.4462 | 0.6751 | 0.0150 | 0.1362 | 2.534 | 0.9384 |
| December 1 | 0.0146 | 4.7527 | 0.6603 | 0.2875 | 7.9066 | 0.6260 | 0.0090 | 0.1509 | 4.357 | 0.8580 |
| December 2 | 0.0144 | 3.5855 | 0.7133 | 0.2424 | 7.8499 | 0.6535 | 0.0093 | 0.1170 | 3.902 | 0.8808 |
| January 1 | 0.0072 | 4.8938 | 0.7511 | 0.1426 | 6.4452 | 0.7145 | 0.0044 | 0.0753 | 4.698 | 0.9159 |
| January 2 | 0.0136 | 21.895 | 0.3838 | 0.2712 | 24.302 | 0.4932 | 0.0043 | 0.2131 | 22.478 | 0.7134 |
| February 1 | 0.0166 | 12.698 | 0.5927 | 0.2459 | 18.913 | 0.4201 | 0.0143 | 0.0530 | 12.841 | 0.7751 |
| February 2 | 0.0171 | 15.445 | 0.6752 | 0.3371 | 19.114 | 0.6422 | 0.0105 | 0.1780 | 14.970 | 0.8682 |
| March 1 | 0.0091 | 26.017 | 0.1685 | 0.2422 | 24.538 | 0.2893 | 0.0004 | 0.2367 | 24.395 | 0.5380 |
| March 2 | 0.0119 | 34.254 | 0.4049 | 0.2596 | 35.352 | 0.4691 | 0.0100 | 0.0799 | 33.259 | 0.6499 |
| | $[O^3]$ = a*elevation + c | | | $[O^3]$ = b*distance + c | | | $[O^3]$ = a*elevation + b*distance + c | | | |

*Also shown are the coefficients for the lineal multiple regression and $r^2$ when both factors (elevation and distance) are considered.

above are different for different periods of the year. In summer and early winter, for example, the $r^2$ is higher (>0.5) than in spring and late winter (Fig. 7). When distance to the city is considered, similar results to the ones found for elevation are observed (Fig. 7). There is a good correlation between the ozone concentration and distance, but not all year. If both factors are combined, the $r^2$ from the multiple linear regression improves substantially (Table 2), but again the correlation is not good for all periods. Correlation with distance is better than correlation with elevation in summer, and the other way around in winter.

## CONCLUSIONS

The results of this study show that the use of passive samplers can help to delimit air pollution zones in relation to ozone in a feasible and cost-effective way in relatively large areas. Around Madrid, concentrations of ozone are higher on the elevated sites at certain distances to the city and urban areas. There is a seasonal cycle, with maximum ozone concentrations occurring in the

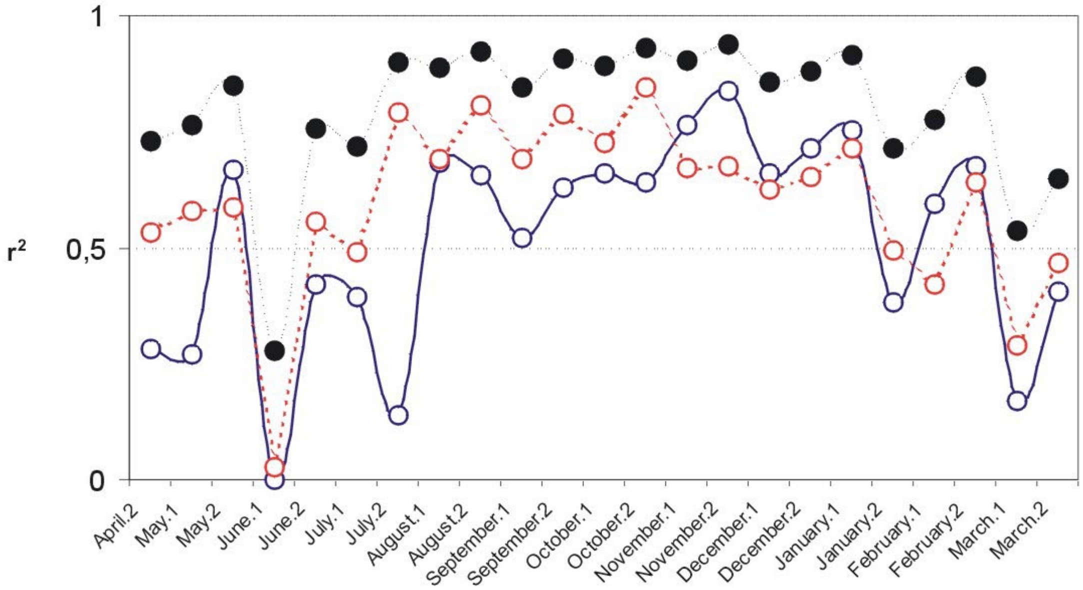

**FIGURE 7**. $R^2$ of the Table 2 regressions throughout the sampling period: distance (red circle), elevation (blue circle), and combination of both (black circle).

basin in late spring or summer depending on the locations. The information obtained allowed us to group the locations into four ozone classes. The ozone concentrations observed in summer and spring can be considered high and likely to produce injury to susceptible vegetation when compared with similar studies in other areas[18,19]. The complex topography of the Madrid Basin can explain the results obtained in this pilot study, which suggests that it would be useful to place one or two continuous ozone monitors at the higher elevations to better interpret the data from the passive samplers. Altitude and distance to the city can explain the observed concentrations during the summer and winter, whereas no single equation can be used to predict ozone values over the year. During the transition periods, however, especially in early spring and to a lesser extent in autumn, there is not a good correlation between elevation or distance. The data from the pilot study strongly suggest that altitudinal gradients for ozone are not always the case.

## ACKNOWLEDGEMENTS

This work has been financed by the DGCN (Ministry of Environment). Since 1999, the Generalitat Valenciana and Bancaixa have supported this group's work on passive sampling. Air quality data were provided by the air quality service of the *Conselleria de Medio Ambiente* (Generalitat Valenciana), with the special help of J.V. Miró. Thanks are given to J.J. Dieguez for his help with the air quality electronic data, and to TECMENA S.L. and ESMA S.L. for helping us with the field work.

## References

1. Naveh, Z., Steinberger, E.H., Chaim, S., and Rotmann, A. (1980) Photo-chemical oxidants: a threat to Mediterranean forest and upland ecosystems. *Environ. Conserv.* **7,** 307–309.

2. Velissariou, D., Davison, A.W., Barnes, J.D., Pfirrmann, T., Maclean, D.C., and Holevas, C.D. (1992). Effects of air pollution on *Pinus halepensis* Mill.: pollution levels in Attica, Greece. *Atmos. Environ.* **26A(3),** 373–380.
3. Gimeno, B.S., Velissariou, D., Barnes, J.D., Inclán, R., Peña, J.M., and Davison, A. (1992) Daños visibles por ozono en acículas de *Pinus halepensis* Miller en Grecia y España. *Ecología* **6,** 131–134.
4. Badot, P.M. and Garrec, J.P. (1993) Dépérissement local du pin d' Alep (*Pinus halepensis*) le long du litoral Méditerranéen. *Rev. For. Fr.* **45**, 134–140.
5. Garrec, J.P. (1994) Le dépérissements littoraux d'abres forestiers. *Rev. For. Fr.* **46,** 454–457.
6. Millán, M.M., Salvador, R., Mantilla, E., and Kallos, G. (1997) Photo-oxidant dynamics in the western Mediterranean in summer: results from European research projects. *J. Geophys. Res.* **102(D7),** 8811–8823.
7. Millán, M.M., Artiñano, B., Alonso, L.A., Castro, M., Fernandez-Patier, R., and Goberna, J. (1993). Meso-meteorological Cycles of Air Pollution in the Iberian Peninsula, (MECAPIP). Air Pollution Research Report 44, (EUR N_ 14834). European Commission DG XII/E-1, Brussels, 1992.
8. Millán, M.M., Salvador, R., Mantilla, E., and Artiñano, B. (1996) Meteorology and photochemical air pollution in Southern Europe: experimental results from EC research projects. *Atmos. Environ.* **30,** 1909–1924.
9. Barry, C. (1964). Differences in concentrations of surface oxidant between valley and mountain conditions in the southern Appalachians. *J. Air Pollut. Control Assoc.* **14,** 238–239.
10. Logan, J.A. (1985). Tropospheric ozone: seasonal behaviour, trends and anthropogenic influence. *J. Geophys. Res.* **90(10),** 10,463–10,482.
11. Georgiadis, T., Giovanelli, G., and Fortezza, F. (1994). Vertical layering of photochemical ozone during land-sea breeze transport. *Nuovo Cimento* **17**, 371–375**.**
12. Puxbaum, H., Gabler, K., Smidt, S., and Glattes, F. (1991). A one-year record of ozone profiles in an alpine valley. *Atmos. Environ.* **25A,** 1759–1756.
13. Aneja, V.P., Li, Z., and Das, M. (1994) Ozone case studies at high elevation in the eastern United States. *Chemosphere* **29,** 1711–1790.
14. Millán, M.M., Mantilla, E., Salvador R., Carratalá, A., Sanz, M.J., Alonso, L., Gangoiti, G., and Navazo, M. (2000). Ozone cycles in the western Mediterranean basin: interpretation of monitoring data in complex coastal terrain. *J. Appl. Meteorol.* **39,** 487–508.
15. Sanz, M.J. and Millán, M.M. (1997). The dynamics of aged air masses and ozone in the western Mediterranean: relevance to forest ecosystems. *Chemosphere* **36(4–5),** 1089–1094.
16. Krupa, S. and Legge, A. (2000). Passive sampling of ambient, gaseous air pollutants: an assessment from an ecological perspective. *Environ. Pollut.* **107,** 31–45.
17. Sally, L.-J., Olson, III, M.P., Allen, G.A., and Koutrakis, P. (1994) Evaluation of the Harvard ozone passive sampler on human subjects indoors. *Environ. Sci. Technol.* **28,** 915–923.
18. Brace, S. and Peterson, D.L. (1998). Spatial patterns of tropospheric ozone in the Mount Rainier region of the Cascade Mountains. *Atmos. Environ.* **32(21),** 3629–3637.
19. Blum, O., Bytnerowicz, A., Manning, W., and Popovicheva, L. (1997) Ambient tropospheric ozone in the Ukrainian Carpathian Mountains and Kiev region: detection with passive samplers and bioindicator plants. *Environ. Pollut.* **98,** 299–304.
20. Pujadas, M., Plaza, J., Terés, J., Artíñano, B., and Millán, M. (2000) Passive remote sensing of nitrogen dioxide as a tool for tracking air pollution in urban areas: the Madrid urban plume, a case of study. *Atmos. Environ.* **34,** 3041–3056.
21. Derwent, R.G. and Davis, T.J. (1994). Modeling the impact of NOx or hydrocarbon control on photochemical ozone in Europe. *Atmos. Environ.* **28,** 2039–2052.
22. Koutrakis, P., Wolfson, J.M., Bunyaviroch, A., Froelich, S.M., Hirano, K., and Mulik, J.D. (1993) Measurements of ambient ozone using a nitrite-coated filter. *Anal. Chem.* **65,** 209–214.
23. Plaza, J. (1997). Caracterización de la Contaminación por ozono en la cuenca áerea de Madrid [Thesis]. Universidad Complutense de Madrid, unpublished.
24. Whitemean, C.D. (1990). Observations of thermally developed wind systems in mountainous terrain. In *Atmospheric Processes over Complex Terrain. Meteorol. Monogr.*, No. 45. American Meteorological Society, Boston, MA. pp. 5–42.

## BIOSKETCH

**Dr. M. J. Sanz** (Biological Sciences, Botany) is Head of Air Pollution Effects Program at Fundación CEAM (Valencia, Spain) and Associated Lecturer of Botany (Universitat de Valencia, Department of Vegetal Biology, Spain). She has spent 14 years in the field of plant vegetation, ecophysiology of plants effects of air pollution and air quality research, and she has over 30 publications. She has been involve in several EU projects dealing with the characterization of the meteorological processes affecting the dynamics of photo-oxidants in the Mediterranean area (SECAP and BEMA II project), their interactions with plant species and communities (including crops, TOMSTRESS project), and energy and $CO_2$ canopy flux measurements in Mediterranean ecosystems (MEDELU and RECAB EU projects). Dr. Sanz also has experience in the field of bioindication, using lichens as well as vascular plants (i.e., EuroBionet Life project). She was member of the Ad-Hoc Working group that drafted the Position Paper on Ozone (EU, 1999), and she participated as a member of the CSTEE group during the draft of the ozone document produce by the committee. She is a Co-Chair of the ICP-Forest Expert Panel on Deposition–WG Ambient Air Quality, mostly dealing with ozone injury evaluation and the use of passive samplers in the Pan-European Program. Her main interests are related to ozone and $CO_2$ canopy fluxes, effects of air pollution, bioindication of air pollution, atmospheric deposition (with special attention to N compounds), and use of passive sampling techniques for air pollution.
